# Gastric Digestion in the Hog

**Published:** 1888-04

**Authors:** Robert Meade Smith


### GASTRIC DIGESTION IN THE HOG.

ROBERT MEADE SMITH, M. D.

The stomach of the hog is generally described as a simple stomach, but it really represents a stage of transition between the simple stomach of carnivora and the complex stomachs of ruminants. Even on external examination, by the presence of constrictions at the cardiac and pyloric extremities, the presence of well marked diverticula is evident. On inspecting the interior, Ellenberger and Hofmeister, in a series of elaborate papers published in recent numbers of the *Archiv fur Wissenschaftliche und Praktische Thierheilkunde*, show that the organ may be divided into five distinct regions. 1. The *œsophageal portion*, which is coated with a mucous membrane, cutaneous in character, similar to that lining the œsophagus, and which is separated by a distinct border from the secreting membrane. It contains no glands, but is papillated, though to a less degree than the left half of the horse's stomach. 2. The *cardiac diverticulum*, lined with a white, thin mucous membrane, which is separated by a fold of mucous membrane from the fundus of the stomach. 3. The *left zone* or *fundus*, constituting one-third or one-half the stomach, lined with a glandular membrane similar to that of the cardiac diverticulum; it contains, however, a relatively smaller number of follicles. 4. The *central zone* includes the greater curvature, and extends towards the pylorus and œsophageal portion, its extremities being triangular. The mucous membrane in this region is very thick and brownish-red in color and contains the so-called fundus glands. These are tubular in structure, longer, but sub-divided to a less degree than the pyloric glands. The mucous membrane of this portion of the hog's stomach is coated with cylindrical epithelium and contains tubular glands, which are shorter than the fundus glands and composed of small, transparent, granular cells, different from those found in any other region. Lymph follicles are numerous, and in some places so close together as to resemble Peyer's patches. The ducts of these tubules are lined with epithelium similar to that lining the cavity of the stomach, while the fundus contains chief and associate cells similar to those found in the stomach of carnivora, with the exception that the associate cells lie in groups external to the cylindrical cells. 5. The *right* or *pyloric zone* includes the pylorus, as much of the lesser curvature as does not belong to the œsophageal portion, and a small portion of the greater curvature. Its mucous membrane is white in color and in the pylorus very thin, though much thicker at its entrance. The submucosa is sparsely developed. After death the pyloric zone is generally found coated with a thick layer of mucus, stained yellowish from the bile. The glands of this portion are considerably longer than those found elsewhere, are subdivided and convoluted. Associate cells are entirely wanting in the pyloric cone, the cells being cylindrical and granular or hyaline.

As Ellenberger and Hofmeister point out, the stomachs of mammals may be divided into two different groups, in one of which œsophageal diverticula, and the other diverticula of the stomach itself, represent the mode of deviation from the simple gastric form. When both forms of diverticula are present the true compound stomach is represented. The gastric formation with a diverticula formed from the glandular stomach itself is met with in many herbivora and omnivora, and such carnivora as live on highly indigestible animal substances.

In its simplest form the part near the Pylorus is dilated into a pouch-like expansion : such a form is met within the hog, but is still further complicated by sacular expansion at the cardiac extremity. Such a formation evidently lengthens the period of retention of the food within the stomach, and by an increase in the internal surface of the stomach permits of a more continuous action of the gastric juice, since it corresponds to an increase in the secreting surface.

In the development of the compound stomach through the formation of œsophageal dilatations, the stomach of the hog represents the first stage.

The second stage is found in the stomach of the horse, where the entire left half of the stomach may be regarded as an œsophageal expansion, and while the stomach of the horse, from external view resembles a simple stomach, internal examination shows that it is practically a compound stomach.

The highest degree of development of the œsophageal pouches is of course seen in the stomach of the ruminants.

The gastric juice of the hog contains the same ferments as is found in this secretion in other mammals ; it dissolves albuminates, and converts them into peptone and para-peptone and syntonin, and coagulates milk, and to a slight degree as in the case of other mammals, splits up fat into glycerine and fatty acids.

The secretion obtained from different portions of the stomach differs: that obtained from the greater curvature contains more mucin, more acid, and more ferment than that from the other portion, while the secretion from the œsophageal portion is free from ferment.

In the secretion from the greater curvature, as obtained by the making of extracts with common salt, the degree of acidity varies from 0.03 to 0.07 per cent.

The portion of the stomach supplied with the associated cells contains all the ferments in the greatest quantity. Small amounts are found in the pyloric region, and a still smaller amount in the cardiac diverticulum.

These ferments in the hog are with difficulty extracted with glycerine, but are readily removed by means of dilute hydrochloric acid and salt solutions.

Ellenberger and Hofmeister further claim that there is a diastatic ferment in the mucous membrane of the hog's stomach, and they have proved by carefully controlled experiments that the conversion of starch into sugar by means of artificial gastric juice from the hog is actually due to the presence of the ferment. It does not, however, seem clear as to whether this ferment is actually produced in the gastric mucous membrane, or whether it enters that membrane by imbibition from saliva which has been swallowed. The gastric juice of the fundus of the stomach of the greater curvature produces coagulation of milk, both in alkaline and in neutral conditions; this power is not possessed by the mucous membrane of the cardiac sac, and only to a slight degree by the pyloric portion. No lactic acid ferment exists in the hog's gastric juice.

The progress of gastric digestion in the hog has been carefully studied by Ellenberger and Hofmeister, by administering definite amounts of specific foods to hogs in whom the stomach had been emptied by fasting and cleansed by copious administration of water. The animals were then killed at specific intervals after the administration of the food, and the gastric and intestinal contents subjected to chemical examination.

These authors' experiments were restricted to the cereals,

The largest number of experiments were made with oats, given dry so as to produce the greatest quantity of saliva. In other instances the food was given moist so as to reduce the secretion of saliva to a minimum. To those who received the dry food, water was always given to drink. The animals on whom these experiments were made were for a few days previous to the experiment fed on such food as could be readily recognized in the intestinal tube. For thirty-six hours before the experiment no food whatever was given. The animals were then killed, after the administration of a weighed quantity of food at one, two, three, four, six, eight, ten and twelve hours after the termination of the meal, an attempt being made to isolate the portions obtained from the different parts of the stomach, the cardiac and pyloric halves being kept separate. The result of these experiments directed attention especially to the progress of digestion, to the location of the gastric contents, the nature of the acid and the quantity of the ferment present, the duration of gastric digestion, the source of gastric juice, and the character of the gastric movements. Their experiments teach that the gastric digestion of grains in the hog may be divided into two periods. During the meal and for one or two hours afterward digestion of starch alone takes place, the starch being converted into soluble starch, dextrin and sugar. Simultaneously with the digestion of the starch lactic acid fermentation occurs, a considerable quantity of the sugar being in this way converted into lactic acid. It does not appear from their results as to whether cellulose also at the same time undergoes fermentation or not. During this period the food becomes softened and swollen, from maceration in the fluids, and a greater part of the soluble matters of the food pass into solution, the digestibility of the vegetable albuminates being facilitated by the presence of lactic acid.

The conversion of the starch into sugar occurring in this period is due to the saliva which the authors have found in the hog to possess amylolytic power in a high degree; it is strongly alkaline, and therefore neutralizes whatever acid was first present in the stomach. Later the cardiac contents become acid, due at first to the presence of lactic acid, which, as is well known, does not interfere with the action of the amylolytic ferment. It is also probable that a certain amount of this amylolytic ferment comes from the cardiac sac of the hog's stomach, which, as already mentioned, contains a special character of glands whose extract always yield diastatic ferments.

As regards the degree of digestion in this period the following figures taken from one of Ellenberger's and Hofmeister's experiments, serve as an example: The animal had received 860 grammes of oats, representing 75 grammes of cellulose, 557 grammes of carbo-hydrates and 93 grammes of albuminoids. As a consequence 22.5 per cent. soluble nutritive substances consisted of albumen. On analysis only 47 grammes of undissolved albumen were found, hence 34 per cent. of the insoluble albuminous bodies had passed into solution.

In feeding with animal matters, naturally this first stage, which might be called the amylolytic period of digestion, is absent. This amylolytic period lasts for about three or four hours, including of course, the half hour or hour occupied by the meal. The duration is, however, longer in the cardiac sac than in the fundus and pyloric portions of the stomach, where it becomes gradually converted into what might be termed the proteolytic period, in which the proteids become converted into peptones.

The digestion of proteids in the hog is first rendered possible through the presence of lactic acids. During this stage of proteolysis the digestive processes differ in the cardiac and pyloric portions of the stomach, indicating that for several hours a well marked difference exists between the contents of the pyloric and œsophageal portions of the stomach; in the former of which only lactic acid and in the latter both hydrochloric and lactic acids are present. This fact, to which we are also indebted to Ellenberger and Hofmeister, is in opposition to the general accepted views as to the rapid diffusion and mixing of the gastric contents.

The second digestive period in which, while the cardiac extremity is still digesting starch, the pyloric half is digesting albumen, begins at about the third or fourth hour of digestion and may continue for nine to twelve hours.

In the third period the digestion of starch ceases, from the great development of hydrochloric acid, although it should be remembered that up to the fourth hour of gastric digestion the cardiac fluid is still capable of converting starch into sugar.

It thus would appear that digestion in the stomach of the hog continues from one meal, to the other, although in a moderate meal, part of the gastric contents may pass into the intestines three or four hours after eating ; but part, nevertheless, remains in the stomach, and may be found there even thirty-six hours afterwards.

These results further show that the degree of mixing of the contents of the stomach produced by the gastric movements is by no means complete, the first quantities being gradually pushed on towards the pylorus by those coming afterwards. The degree of acidity of the gastric contents gradually increases : while at first alkaline it gradually increases in acidity so that three hours after the meal 0.07 per cent. acid may be recognized in the left half of the stomach and 0.2 in the right half. The acid within the left half of the stomach then commences to increase until finally it may amount to 0.3 per cent. When the meals rapidly follow each other gastric digestion is interrupted and the undigested portion is forced into the intestine, undergoing digestion there by means of the intestinal digestive fluids. As a consequence, in large meals the amylolytic period is increased and the proteolytic period decreased, while the degree of acidity more slowly approaches a maximum. The degree of mastication is further of influence on the completeness of the amylolytic change. When dry food is given mastication is more prolonged and the conversion into starch is more complete than when soft watery foods are given.

In the hog, therefore, gastric digestion is most closely analogous to gastric digestion in the carnivora, but it is slower and less complete. Thus Colin states that after giving one thousand grammes of raw meat to a hog, six hundred grammes were found in the stomach six hours after feeding while undigested pieces were found in the small intestines. In another case a hog which had received three kilo of meat with one litre of water, had digested only three hundred grammes in six hours. We thus see that while the hog is an omnivorous animal it is not less capable of digesting animal matters than are the pure carnivorous animals. While this is, however, the case from their imperfect mastication, they are less constituted for the extracting of nutritive principal from the vegetable matter than the purely herbivorous animals.

---

THE UNITED STATES VETERINARY MEDICAL ASSOCIATION.—The semi-annual meeting of this Association in Baltimore, on March 20th, was unanimously acceded to be, both scientifically and socially, the most successful one that has been held for some years. From the entrance of the visiting members into the Monumental City until the regretted moment of their departure, they were the recipients of the courtesy and hospitality which Baltimore is celebrated for, and Dr. Dougherty and his colleagues will long be remembered most gratefully. The business portion of the meeting was conducted with precision and rapidity by Dr. Miller, leaving the entire afternoon for the papers of Drs. Salmon and Clement, which were a scientific treat. We will borrow the expression of a prominent medical man, who said that "it was with surprise that he had learned that his profession would have to come to the veterinarians for a better study of pathology.

---

LEGISLATION FOR AN ARMY VETERINARY SERVICE.—That there is great need for this, no one, veterinarian or agriculturist, legislator or military man, denies. The bill which has just been introduced in the House of Representatives will undoubtedly do much good, even if it only acts as an entering wedge. Prominent men in the army recognize the necessity of a proper veterinary service, and will aid in obtaining it at the right moment. But judgment should be used in framing an act which is of serious importance to the personel of the Army, and those who are directly interested in it should be consulted. This was not done in the present case. A dozen long articles are introduced where three would suffice. In this Act of Congress, which should merely establish a corps, are embodied many regulations which already exist in the Army Statutes ; there are added many regulations and details which interfere or are opposed to the present discipline, or which would seriously hamper and incommode the details of the service which should be free to make its own regulations. In its present form the bill cannot possibly pass, but we hope the proper modifications may be made, and that the representative of every reader of THE JOURNAL will vote for it.

---

CATTLE QUARANTINE IN PENNSYLVANIA.—The Pennsylvania authorities have at last, with the slowness characteristic with that Commonwealth, followed the example of other States, and have consented to co-operate with the General Government in the extirpation of the Lung-plague. His Excellency, Governor Beaver, issued the proclamation on March 23d. There are but few cattle near the centre of the city of Philadelphia, but the eight-mile radius takes in the stock-yards, the large slaughter houses, and reaches important dairy districts on the border of the city in two other counties. This area is certainly the most suspicious in Pennsylvania, and the result of the inspection will show if it is worth while to go further. The exact condition of the cattle stables has never been known as the State Inspector visits only those animals which are reported by their owners, we trust that the State may be as free from Contagious Pleuro-pneumonia as it has been reported to be.

